# Sensitization pattern to environmental allergens in a Japanese population

**DOI:** 10.1016/j.jacig.2022.10.004

**Published:** 2022-12-13

**Authors:** Asako Kitahara, Yoshiro Yamamoto, Yuma Fukutomi, Yoshiki Shiraishi, Jun Tanaka, Tsuyoshi Oguma, Masami Taniguchi, Tadashi Nagai, Koichiro Asano

**Affiliations:** a Division of Pulmonary Medicine, Department of Medicine, Tokai University School of Medicine, Kanagawa, Japan; b Department of Mathematics, School of Science, Tokai University, Kanagawa, Japan; c Clinical Research Center, National Hospital Organization Sagamihara National Hospital, Kanagawa, Japan; d Shonan Kamakura General Hospital Center for Immunology and Allergology, Kanagawa, Japan; e Central Blood Institute, Japanese Red Cross Society, Tokyo, Japan

**Keywords:** Allergen, cluster analysis, factor analysis, general population, IgE

## Abstract

**Background:**

We previously described the prevalence of allergen-specific IgE in a general population of Japanese adults.

**Objective:**

We sought to elucidate allergen sensitization patterns in this population.

**Methods:**

Serum samples had been obtained from 800 blood donors aged 20 to 59 years and living in Tokyo, Japan, in 2005 and stored in the Japanese Red Cross Society. These samples were examined for IgE levels, total and specific for 23 allergens or allergen sources correlated with allergic airway diseases using the ImmunoCAP method. Exploratory and confirmatory factor analyses were performed to uncover the relationship among allergen-specific IgE based on their titers. Hierarchical cluster analysis was executed using Ward’s method based on standardized factor scores identified through factor analysis.

**Results:**

Exploratory factor analysis revealed 6 categories of allergen-specific IgE: specific to 2 types of animals (insects and *Dermatophagoides pteronyssinus*/animal dander), 2 types of pollens (group 1 [Japanese cedar and cypress] and group 2 [alder, grass, and weeds]), and 2 types of microorganisms (fungi and commensal microorganisms on the skin). The Japanese population was categorized into 3 clusters: (A) nonatopic type, (B) house dust mite–dominant sensitization type, and (C) panatopic type. The panatopic group could be further classified into 2 subclusters positive and negative for fungal sensitization.

**Conclusions:**

This study demonstrated that a Japanese population could be divided into 3 clusters according to the sensitization pattern to 6 types of allergens.

Sensitization to specific allergens is an initial step for the development of allergic diseases. The prevalence of allergic sensitization and the type of allergens sensitized in a general population vary depending on ethnic and environmental factors, such as geographic region and climate.^1^, ^2^, ^3^, ^4^ Therefore, the epidemiologic data of allergen sensitization patterns obtained in a specific population or region are useful for the clinical practice of allergic diseases. A survey in the United States demonstrated that house dust mite (HDM), perennial rye, and German cockroach are the most common aeroallergens that sensitized subjects aged 6 to 59 years.^5^ The European Community Respiratory Health Survey revealed that HDM (*Dermatophagoides pteronyssinus*), grass pollen, and cat allergen are the most prevalent.^1^ Our previous study examined the serum levels of specific IgE to 17 aeroallergens among 800 Japanese healthy blood donors aged 20 to 59 years and demonstrated that the sensitization rate is the highest for Japanese cedar, followed by cypress, *D pteronyssinus*, and moth.^6^

In addition to information on sensitization to an individual allergen, the pattern of sensitization is clinically relevant to disease manifestation. Polysensitization to allergens is correlated with the severity of allergic diseases.^7^^,^^8^ Allergen sensitization patterns are influenced by several factors, including environmental factors, such as temperature, humidity, hygiene conditions, exposure to animals, socioeconomic status, and host factors, including genetic predisposition.^9^ They are also affected by age. Sensitization to HDM and other indoor allergens during childhood shows 3 major patterns: sensitization to HDM alone, cosensitization to HDM and other inhaled allergens, and cosensitization to HDM and food allergens.^10^ Cosensitization to HDM and food allergens mostly occurs during infancy, whereas sensitization to HDM alone or a combination of HDM and inhaled allergens increases depending on age.^10^ The shift of allergen sensitization patterns is clinically associated with variations in prevalent diseases from food allergy/eczema to asthma/allergic rhinitis known as allergic march.^11^^,^^12^

In the present study, we performed an extended analysis of the data of 23 allergen-specific IgE correlated with allergic airway diseases in 800 Japanese healthy blood donors^6^ to clarify the allergen sensitization patterns in the general population of Japan. With factor and cluster analyses, we identified 6 categories of allergen-specific IgE and 3 clusters and 6 subclusters of the population with different allergen sensitization patterns.

## Methods

### Subjects

As previously reported,^6^ 800 samples, 100 per age and sex–stratified group (4 age groups: 20-29, 30-39, 40-49, 50-59 years; 2 genders: male or female) were randomly selected among the serum obtained from blood donors in Tokyo, Japan, in October 2005 and stored at −30°C for 12 years in Japanese Red Cross Society to screen the blood products’ safety. The samples positive for the antibodies or nucleotides of hepatitis B virus, hepatitis C virus, or HIV were excluded before the selection.

This study was approved by the Institutional Review Board of Tokai University Hospital (16R-106) and Sagamihara National Hospital (2016-027) and implemented in compliance with the Declaration of Helsinki. Informed consent was obtained in the form of an opt out on the website of Japan Red Cross Society (https://www/jrc.or.jp/english/). Those who rejected were excluded.

### Measurement of total and allergen-specific IgE

Total and allergen-specific IgE levels were determined using an ImmunoCAP system (ThermoFisher Scientific, Uppsala, Sweden). The IgEs specific for crude extract of 23 allergens or allergen sources were examined: grass pollen (orchard grass/cocksfot [g3]), weed pollens (common ragweed [w1], mugwort [w6], Japanese hop [w22]), tree pollens (alder [t2], Japanese cedar [t17], cypress [t24]), animal dander (dog dander [e5] and cat dander [e1]), *D pteronyssinus* (d1), insects (cockroach [i6], chironomid [bloodworm] [i7], moth [i8]), airborne fungi (*Aspergillus fumigatus* [m3], *Alternaria alternata* [m6], *Cladosporium herbarum* [m2], *Penicillium chrysogenum* [m1]), and commensal bacteria/fungi (*Staphylococcus aureus* enterotoxin A [SEA, m80], *S aureus* enterotoxin B [SEB, m81], toxic shock syndrome toxin-1 [TSST-1, m226], *Candida albicans* [m5], *Malassezia* spp. [m227], and *Trichophyton rubrum* [m205]). An allergen-specific IgE level of greater than or equal to 0.35 U_A_/mL was defined as positive.

### Statistical analyses

Numerical data were expressed as the mean and SD or median and interquartile range (IQR). Categorical data were presented as numbers and percentages. Exploratory factor analysis was performed to classify 23 allergen-specific IgEs according to log-transformed IgE titers. If the value was 0, a value of 0.001 was substituted. Parallel analysis, the Bayesian information criterion (BIC), and BIC considering the sample size were used to determine the optimal number of factors. Confirmatory factor analysis was conducted to examine how the allergen groups were correlated with each other. Cluster analysis was performed on the basis of the standardized factor scores. The trend analysis between increasing age categories and allergen-specific IgE levels was conducted via the Jonckheere-Terpstra test. Differences in categorical and numerical data among the clusters were analyzed using chi-square and Kruskal-Wallis tests, respectively. Data with *P* less than .05 were considered significant. Statistical analyses were performed using R with psych and lavaan packages.

## Results

### Factor analysis of allergen-specific IgE

The exploratory factor analysis based on IgE titers was performed to determine the number of factors classifying the 23 allergen-specific IgE. Through parallel analysis, 6 factors were identified. Seven and 8 factors were proposed by BIC criteria and individual BIC, respectively; however, these models yielded more than 1 allergen-specific IgE contributing to multiple factors, or none contributing to a specific factor. Therefore, subsequent analyses were performed using the 6 factors identified by parallel analysis, in which each allergen belongs to a single factor (see Table E1 in this article’s Online Repository at www.jaci-global.org). There were 2 factors specific to animal-derived allergens (insects and *D pteronyssinus*/animal dander), 2 factors specific to pollen allergens (group 1 [Japanese cedar and cypress] and group 2 [alder, grass, and weeds]), and 2 factors specific to allergens derived from microorganisms (fungi [except for *Malassezia* spp*.*] and commensal microorganisms on the skin [*S aureus* and *Malassezia* spp*.*]) (Table E1).

The prevalence of sensitization to at least 1 allergen (Table I) was the highest for group 1 pollens (Japanese cedar and cypress, 66.8%), followed by *D pteronyssinus*/animal dander (40.1%), group 2 pollens (35.0%), and insects (32.1%). The sensitization rates of *D pteronyssinus*/animal dander, insects, and airborne fungi/*Trichophyton* were significantly higher in men than in women (*P* < .01-.001). The sensitization rates significantly differed among the age groups at least in either gender except for the group of airborne fungi/*Trichophyton*; younger subjects tended to be sensitized more frequently than the older population did (Table I; see Fig E1 in this article’s Online Repository at www.jaci-global.org).

**TABLE I:** Sensitization rate to allergen types in each sex and age group

Allergen type	Sex	Sensitization rate (%)						
		All subjects	Divided by sex	Divided by sex and age group (y)				*P*∗
				20-29	30-39	40-49	50-59	
*Dermatophagoides pteronyssinus*/ animal dander	Male	40.1	46.5†	60	55	38	33	<.001
	Female		33.8	53	29	28	25	<.001
Insects	Male	32.1	37.3‡	39	42	32	36	NS
	Female		27.0	32	32	24	20	.03
Group 1 pollen	Male	66.8	68.8	80	69	68	58	.001
	Female		64.8	65	67	69	58	NS
Group 2 pollen	Male	35.0	38.3	55	49	22	27	<.001
	Female		31.8	37	36	25	29	NS
Fungi	Male	13.6	16.8‡	17	13	17	20	NS
	Female		10.3	13	13	7	8	NS
Commensal microorganisms on the skin	Male	16.6	17.3	23	19	13	14	NS
	Female		15.8	28	8	15	12	.01

*NS*, Not significant.

∗ Trend analysis for age categories with the Jonckheere-Terpstra test.

† *P* < .001.

‡ *P* < .01.

**FIG 1:**
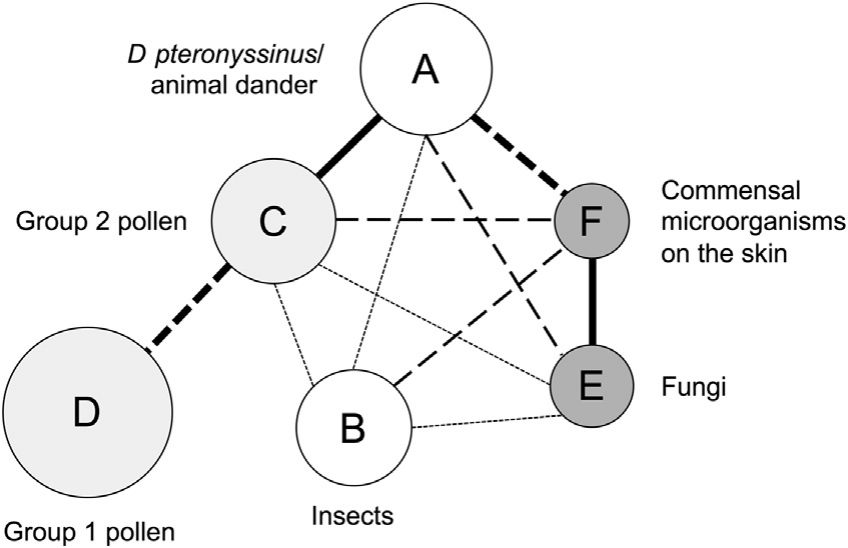
Confirmatory factor analysis demonstrating the relationship among 6 factors for allergen-specific IgE. The area of circles represents the sensitization rate of specific factors. Bold solid lines suggest the strongest interaction between the factors (*r* ≥ 0.75), followed by a bold dashed line (0.70 ≤ *r* < 0.75), a dashed line (0.65 ≤ *r* < 0.70), and dotted lines (0.60 ≤ *r* < 0.65).

Confirmatory factor analysis was performed to evaluate the correlation between the factors of allergen-specific IgEs (Fig 1). The specific IgE levels for each allergen were correlated with the standardized scores of the designated factor with *r* of greater than or equal to 0.70 except for TSST-1 (*r* = 0.69) and *A alternata* (*r* = 0.61) (see Table E2 in this article’s Online Repository at www.jaci-global.org). The correlation between the 2 factors of microorganism-derived allergen-specific IgEs and between the 2 factors of pollen-derived allergen-specific IgEs was high (*r* = 0.78 and 0.71, respectively); conversely, the correlation between sensitizations to the 2 animal-derived allergen groups was moderate (*r* = 0.64). The correlation between sensitization to *D pteronyssinus*/animal dander and group 2 pollens (*r* = 0.76) or commensal microorganisms on the skin was also high (*r* = 0.74). Sensitization to group 1 pollens was relatively independent of sensitization to other allergen groups except for group 2 pollens.

### Cluster analysis

**FIG 2:**
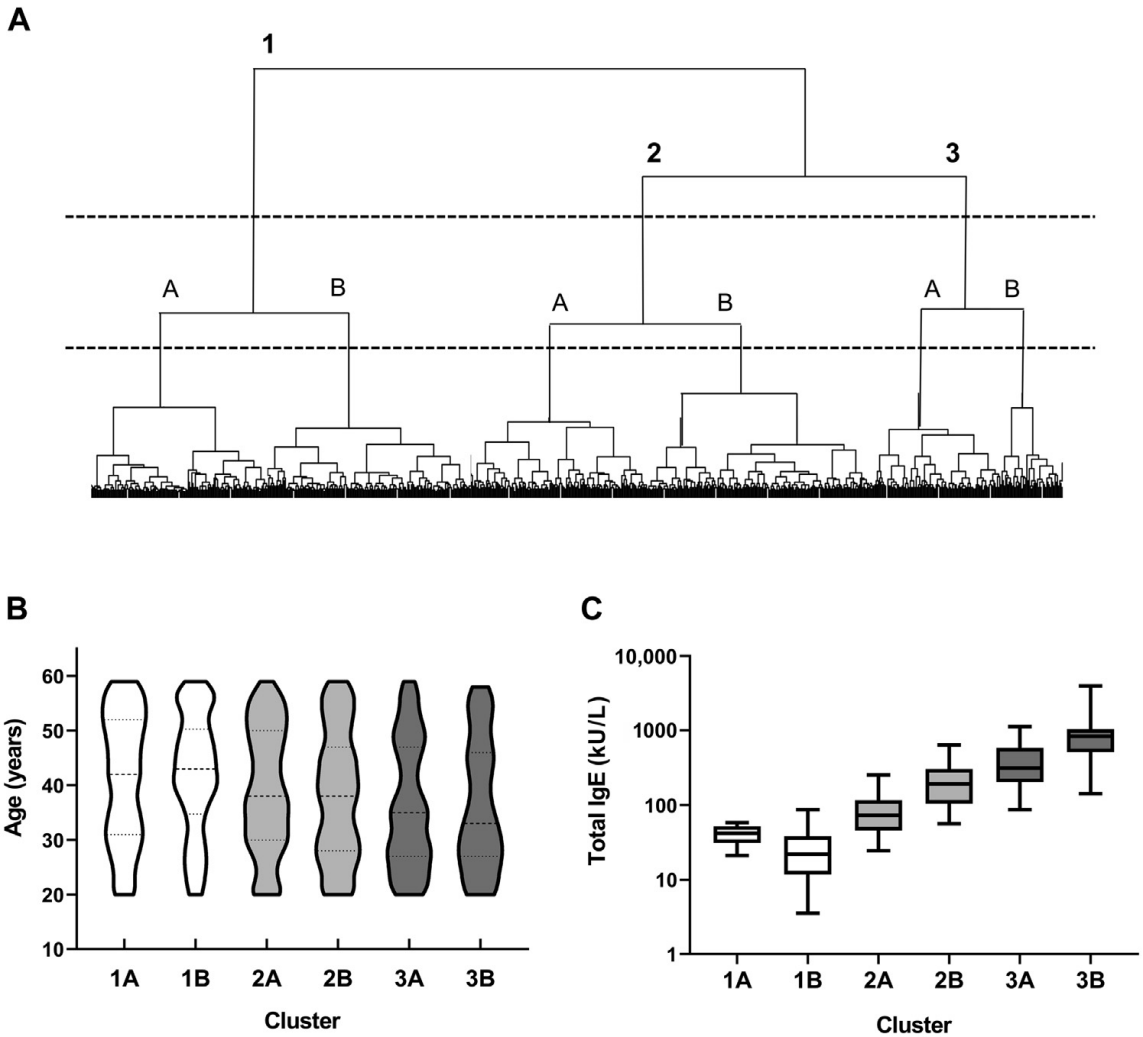
Three clusters identified by Ward’s method. **A,** A cluster dendrogram. Three clusters were identified, and each of them had 2 subclusters. Violin plots of age (**B**) and total IgE levels in serum (**C**) in the 6 clusters/subclusters.

Cluster analysis was then performed to clarify the characteristics of each individual’s sensitization pattern using Ward’s method. Analysis was performed using 6-factor scores instead of IgE titers for each allergen to make it easier to comprehend the characteristics of the allergen sensitization patterns for each cluster. Because there was a large variation among the SD of factor scores (0.40-1.25), the scores were standardized before analysis. According to the cluster dendrogram in our study, 3 major clusters, each having 2 subclusters, were identified (Fig 2, *A*). Age and sex significantly differed among the clusters (*P* < .05-.001). In cluster 1 (n = 317), 60.9% of the subjects were older than 40 years, and women were slightly predominant (54.9%). Conversely, 64.1% of the subjects in cluster 3 (n = 153) were younger than 40 years, and men were predominant (58.2%; Fig 2, *B*). Cluster 2 (n = 330) tended to be in the middle of clusters 1 and 3. The median total IgE levels in serum were 32 kU/L (IQR, 20-48) for cluster 1, 104 kU/L (IQR, 60-195) for cluster 2, and 451 kU/L (IQR, 241-841) for cluster 3 (*P* < .0001; Fig 2, *C*).

The number of sensitized allergens and sensitization rates to the 6 types of allergens for each cluster/subcluster are presented in Table II and Fig E2 in this article’s Online Repository at www.jaci-global.org. Cluster 1 exhibited the lowest rate of sensitization to most allergen types (<20%) except for sensitization to group 1 pollen allergen in subcluster 1B (59.4%). The rate of sensitization to pollen allergens (1A < 1B) and insect allergens (1A > 1B) significantly differed between subclusters 1A and 1B. The subjects in clusters 2 and 3 were paucisensitized (2-4 sensitization) and polysensitized (5 or more sensitization), respectively.^13^ The sensitization rates of subcluster 2B to the pollen and microorganism-derived allergens were significantly higher than those of subcluster 2A; conversely, the sensitization rates of subcluster 3B were significantly higher than those of subcluster 3A only to the microorganism-derived allergens.

**Table II:** Number of sensitized allergens and sensitization rate to the allergen type for each cluster/subcluster

Cluster (n)	1 (317)		2 (330)		3 (153)	
Subcluster (n)	1A (147)	1B (170)	2A (186)	2B (144)	3A (105)	3B (48)
No. of sensitized allergens∗	0.9 ± 1.1		3.5 ± 1.6		8.6 ± 3.5	
	0.4 ± 0.8	1.3 ± 1.2	2.8 ± 1.3	4.3 ± 1.5	7.5 ± 2.4	11.0 ± 4.1
Sensitization rate (%)						
Any allergen	51.7		97.0		100	
	33.3	67.6	95.2	99.3	100	100
*Dermatophagoides pteronyssinus*/ animal dander	12.9		42.7		90.8	
	12.2	13.5	33.3	54.9†	90.5	91.7
Insects	9.8		30.3		81.5	
	15.6†	5.3	27.4	34.0	84.8	75.0
Group 1 pollen	34.1		87.0		90.8	
	4.8	59.4†	83.8	92.4†	92.4	87.5
Group 2 pollen	10.4		39.4		69.9	
	2.0	17.6†	34.9	52.1†	70.5	68.8
Fungi	3.2		10.6		41.2	
	4.1	2.4	5.4	17.4†	24.8	79.2†
Commensal microorganisms on the skin	2.8		15.8		47.1	
	3.4	2.4	8.6	25.0†	30.5	85.0†

∗ Mean ± SD.

† *P* < .05.

The subjects in subcluster 3B were generally young (Fig 2, *B*); however, the median age of those sensitized to microorganism-derived allergens was different according to the type of microorganism (see Fig E3 in this article’s Online Repository at www.jaci-global.org). Sensitization to *A alternata* was more common in younger populations (*P* < .05), and a similar trend was observed for the sensitization to other airborne fungi such as *A fumigatus* and *C herbarum*. However, the sensitization to fungi colonized on the skin or mucosal surface (*T rubrum*, *Malassezia* spp., and *C albicans*) was observed in all age ranges, and sensitization to *S aureus*–derived toxins, SEA and SEB, was more common in older populations, although this was not statistically significant.

## Discussion

In the present study, we classified 23 allergen-specific IgEs that are associated with allergic airway diseases based on the titers in a Japanese population. Six overall categories were identified to classify the allergen-specific IgEs. In addition, the cluster analysis of the studied population identified 3 clusters and 6 subclusters with different atopic statuses and patterns of allergen sensitization.

The present study identified 6 allergen categories. In addition to HDM, animal dander, fungi, and pollen that have been reported in previous studies,^13^, ^14^, ^15^, ^16^ we identified insects and skin commensal microorganisms as independent allergen categories that the general population is sensitized to. There are several reasons for polysensitization to the allergens in a specific category. For example, each kingdom has specific panallergens, such as tropomyosins for animal-derived allergens, profilins, polcalcins, nonspecific lipid transfer proteins, pathogenesis-related protein family 10 members for plants, and manganese superoxide dismutase, cyclophilins, and enolases for fungi.^17^ Factor analysis revealed that the 6 allergen categories could be divided in terms of kingdom (Animalia, Plantae, or Fungi/Bacteria), suggesting that the polysensitization observed in this population was partially caused by cross-reactivity to panallergens.^18^

Another reason is that people are exposed to allergens in certain environments. *D pteronyssinus*, animal dander, and some insects (cockroaches, booklice, and some type of moth) are mainly indoor allergens that can sensitize infants; conversely, pollen allergens are present in outdoor environments, and sensitization to these allergens occurs in late childhood or even later in adulthood. This hypothesis was supported by Yamamoto et al,^19^ who examined children aged 5 and 9 years. They found that the sensitization rate for indoor allergens is consistent regardless of age, whereas older children are more frequently sensitized to outdoor allergens than to indoor allergens.

For microorganism-derived allergens, the site of allergens is an important factor that determines sensitization patterns. *Malassezia* and *S aureus* primarily colonize mostly the skin and the nasal mucosa; they modify the pathobiology of atopic dermatitis and chronic rhinosinusitis with immunologic and nonimmunologic mechanisms.^20^^,^^21^ Furthermore, the presence of IgE to *S aureus*–derived superantigens such as TSST-1 and SEA/SEB, but not *S aureus* carriage, has been demonstrated to predict the subsequent development of severe asthma.^22^^,^^23^ Conversely, *A fumigatus*, *P chrysogenum*, *A alternata*, and *C herbarum* are airborne allergens in indoor/outdoor environments. *Trichophyton* is the pathogen causing dermatophytosis, a fungal infection of the skin, hair, and nails; however, sensitization to *T rubrum* is associated with asthma severity.^24^ It has been classified with other fungi but not with commensal microorganisms on the skin. It suggests that *T rubrum* from the skin debris of patients with tinea corporis may be inhaled as bioaerosol,^25^ or cross-reactivity may occur between *T rubrum* and other airborne fungi. Ohn et al^16^ classified *C albicans*, another commensal fungus on the mucosal surface, into the group of fungi although its association with this group was modest (*r* = 0.39) and weakly associated with the cutaneous commensal microorganism group (*r* = 0.28).

Cluster analysis based on the standardized factor scores identified 3 clusters: cluster 1 as nonatopic or hypoatopic phenotype; cluster 2 as paucisensitized phenotype predominantly to pollen; *D pteronyssinus*, animal dander, and insect allergens; and cluster 3 as panatopic phenotype. Previous studies also identified hypoatopic and panatopic phenotypes in a birth cohort of Europe^26^ or patients with asthma^27^ and HDM- and pollen-dominant phenotypes, which are indistinguishable in our study. We could not discriminate *D pteronyssinus*/animal dander– and pollen-dominant phenotypes possibly because of 2 reasons. One is the lack of a pediatric population in our study because the samples were obtained from blood donors from the Japanese Red Cross Society, who must be 16 years or older. Younger children, especially during infancy, are exposed mostly to indoor allergens, such as HDM and animal dander, not to outdoor allergens, such as pollen; conversely, the positivity rate to pollen sensitization increases according to exposure length.^28^ Therefore, a significant proportion of the subjects with *D pteronyssinus*/animal dander–dominant sensitization was also sensitized to pollen allergens in our study. Another reason is the high level of group 1 pollen (Japanese cedar and cypress) allergens in Japan, causing almost ubiquitous sensitization (87.0%) in cluster 2 and difficulty in distinguishing between HDM- and pollen-dominant phenotypes.

Another important finding in our study is the identification of subclusters, including subcluster 1B mostly monosensitized to group 1 pollen. Cluster 3B is especially unique, characterized by a higher sensitization rate to microorganism-derived allergen groups than to other groups. There was, however, a substantial difference in the sensitization pattern to airborne fungi and commensal microorganisms. The sensitization to airborne fungi, especially to *A alternata*, was more common in the younger population, whereas there was no such age dependence in the sensitization rate to commensal microorganisms such as *C albicans* and *S aureus*, as we previously demonstrated in the patients with asthma.^29^ Advanced age is, however, associated with a higher prevalence of sensitization to *S aureus*–derived toxins in the general population and patients with asthma.^30^^,^^31^ We also found that the sensitization rate of *S aureus*–derived toxins (TSST-1, SEA, SEB) was 77.8% in the subjects 40 years or older in cluster 3B and 60.0% in those younger than 40 years. Further studies on age-dependent dysbiosis would clarify the mechanisms of this phenomenon.

The present study has several limitations. Although we had attempted to minimize sampling bias by randomly selecting serum stored in the Japan Red Cross Society, the subjects tested were limited to residents in Tokyo. In fact, our previous study has demonstrated that sensitization to pollen allergen is significantly different according to geographic region.^6^ However, the approach we used to classify the study population on the basis of the standardized factor scores for allergen-specific IgE, instead of individual IgE titers for each allergen, may allow our results to be more generalizable to other populations or regions. Second, as previously discussed, serum samples from subjects younger than 16 years or older than 59 years were not available from the source we used to obtain the samples.

### Conclusions

In our study, allergen-specific IgEs can be divided into 6 categories based on different origins and localizations of allergens. A general population can be classified on the basis of not only atopic predisposition but also the type and number of sensitized allergens. Further studies are required to identify environmental and host factors, such as genetic components and gut microbiome associated with specific allergen sensitization patterns.

**Clinical implications**

The general population and patients with allergic airway diseases can be categorized into hypoatopic, paucisensitized, and panatopic phenotypes according to sensitization patterns to allergens; the knowledge of these phenotypes may aide in accurate diagnosis of these diseases and allow for more effective immunotherapy.
