# Supplementary material for: Sensitization pattern to environmental allergens in a Japanese population
Source: J Allergy Clin Immunol Glob. 2022 Dec 13;2(1):30–5. doi: 10.1016/j.jacig.2022.10.004 (PMC10509945; doi:10.1016/j.jacig.2022.10.004)
Supplement: Supplementary Files [file mmc1.pdf]

Supplemental materials

Sensitization pattern to environmental allergens in a Japanese population

Asako Kitahara, MD[1], Yoshiro Yamamoto, PhD[2], Yuma Fukutomi, MD[3], Jun Tanaka, MD[1], Yoshiki Shiraishi, PhD[1], Tsuyoshi Oguma, MD[1], Masami Taniguchi, MD[3,4], Tadashi Nagai, MD[5], Koichiro Asano, MD[1, *]

*Corresponding author

[1]Division of Pulmonary Medicine, Department of Medicine, Tokai University School of Medicine, Kanagawa, Japan

[2]Department of Mathematics, School of Science, Tokai University, Kanagawa, Japan

[3]Clinical Research Center, National Hospital Organization Sagamihara National Hospital, Kanagawa, Japan

[4]Shonan Kamakura General Hospital Center for Immunology and Allergology, Kanagawa, Japan

[5]Central Blood Institute, Japanese Red Cross Society, Tokyo, Japan

Supplemental Table I. Six classifying factors identified by parallel analysis

Name of allergen	Classifying factors of allergen-specific IgE					
	A	B	C	D	E	F
Dermatophagoides pteronyssinus	0.64	0.21	-0.06	0.00	-0.08	0.07
Cat dander	0.95	-0.09	0.06	-0.02	-0.03	-0.02
Dog dander	0.8	0.02	0.21	-0.07	-0.01	0.03
Cockroach	-0.02	0.95	0.02	-0.01	0.02	-0.06
Chironomid	0.02	0.85	0.01	0.02	0.00	0.06
Moth	0.02	0.94	-0.03	0.01	-0.01	-0.06
Grass pollen	0.12	-0.09	0.79	0.05	-0.03	-0.09
Alder pollen	0.06	-0.06	0.67	0.06	0.01	-0.06
Common ragweed	-0.04	0.06	0.84	-0.04	0.01	0.00
Japanese hop	-0.05	0.06	0.79	-0.01	0.05	0.14
Mugwort	-0.05	0.12	0.83	-0.02	-0.01	0.11
Japanese cedar	-0.01	-0.04	0.09	0.89	-0.05	0.04
Cypress	-0.07	0.07	0.10	0.94	0.01	-0.01
Penicillium chrysogenum	-0.08	0.01	0.04	-0.05	0.92	0.00
Cladosporium herbarum	0.05	0.02	0.01	0.02	0.67	0.16
Aspergillus fumigatus	0.04	-0.10	-0.06	0.00	0.94	-0.06
Alternaria alternata	0.28	-0.01	0.01	0.07	0.49	-0.14
Trichophyton rubrum	-0.22	0.07	0.04	-0.04	0.89	-0.03
Candida albicans	0.06	0.08	-0.02	0.01	0.39	0.28
Malassezia sp.	0.22	-0.01	-0.02	0.01	0.24	0.43
TSST-1	-0.03	-0.05	0.02	0.00	-0.05	0.79
SEA	-0.01	-0.03	-0.01	0.01	0.02	0.84
SEB	-0.01	0.03	-0.02	0.02	0.00	0.78

Each number indicates the correlation coefficient.

SEA, *Staphylococcus aureus* enterotoxin A; SEB, *Staphylococcus aureus* enterotoxin B; TSST-1, toxic shock syndrome toxin-1

Supplemental Table II. Correlation coefficient of allergen-specific IgE within the designated classifying factor (confirmatory factor analysis)

A	*r*	B	*r*	C	*r*
Dermatophagoides pteronyssinus	0.71	Cockroach	0.91	Alder	0.67
Cat dander	0.85	Chironomid (bloodworm)	0.92	Grass	0.71
Dog dander	0.96	Moth	0.89	Common ragweed	0.83
				Mugwort	0.92
				Japanese hop	0.91

D	*r*	E	*r*	F	*r*
Japanese cedar	0.92	*Trichophyton rubrum*	0.70	SEA	0.80
Cypress	1.01	*Penicillium chrysogenum*	0.85	SEB	0.78
		Aspergillus fumigatus	0.82	TSST-1	0.69
		Cladosporium herbarum	0.85	*Malassezia sp.*	0.78
		Alternaria alternata	0.61		
		Candida albicans	0.70		

SEA, *Staphylococcus aureus* enterotoxin A; SEB, *Staphylococcus aureus* enterotoxin B; TSST-1, toxic shock syndrome toxin-1

Supplemental Figure 1. Sensitization rate to allergen types in each gender and age group

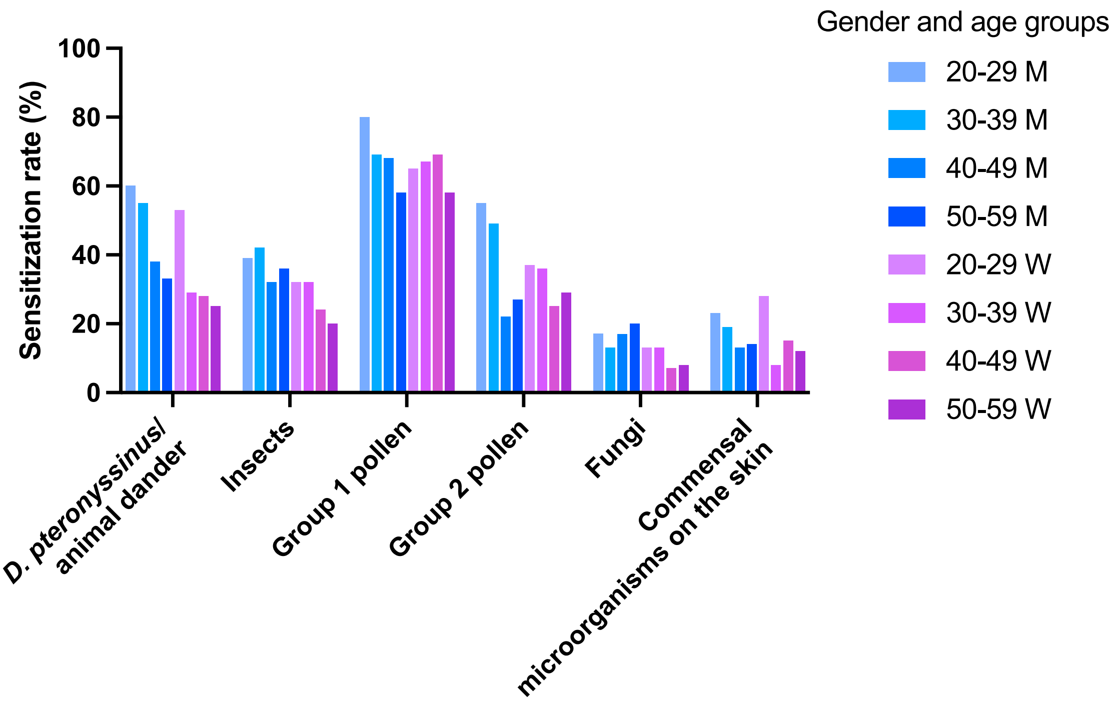

Sensitization rate to six types of allergens in men (M) and women (W) are demonstrated according to each age group (20-29, 30-39, 40-49, 50-59 years).

Supplemental Figure 2. Sensitization rate to the allergen types for each cluster/subcluster

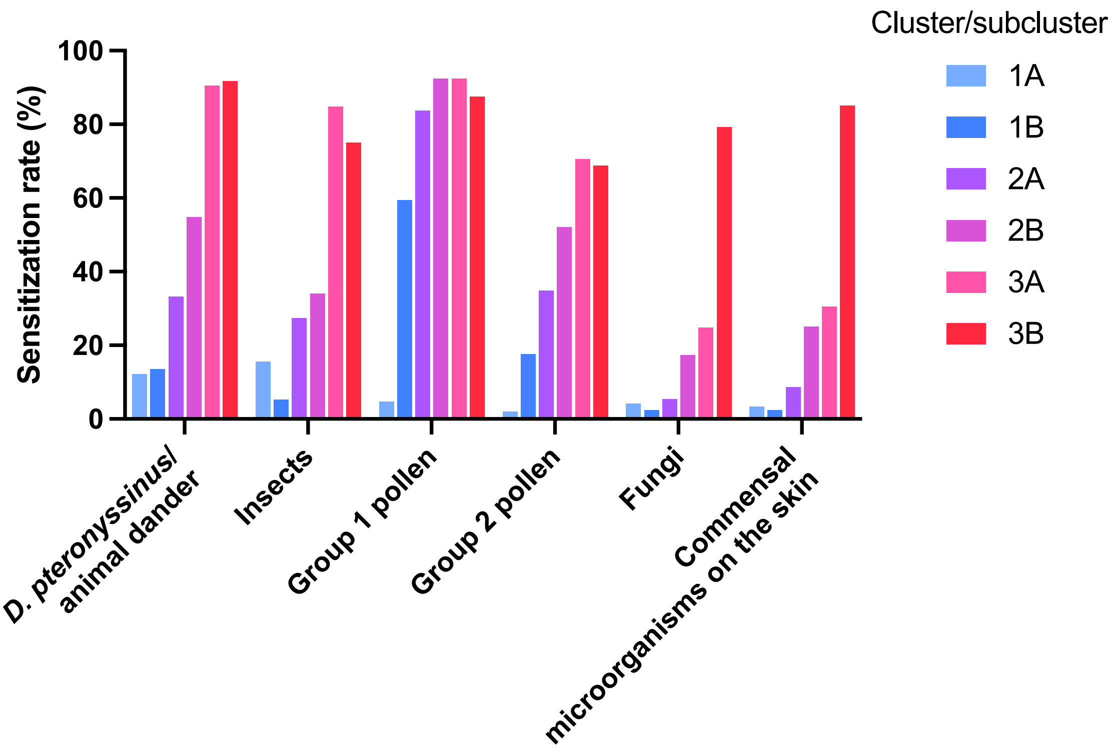

Supplemental Figure 3. Age distribution of the subjects in cluster 3B according to the type of microorganism-derived allergens sensitized

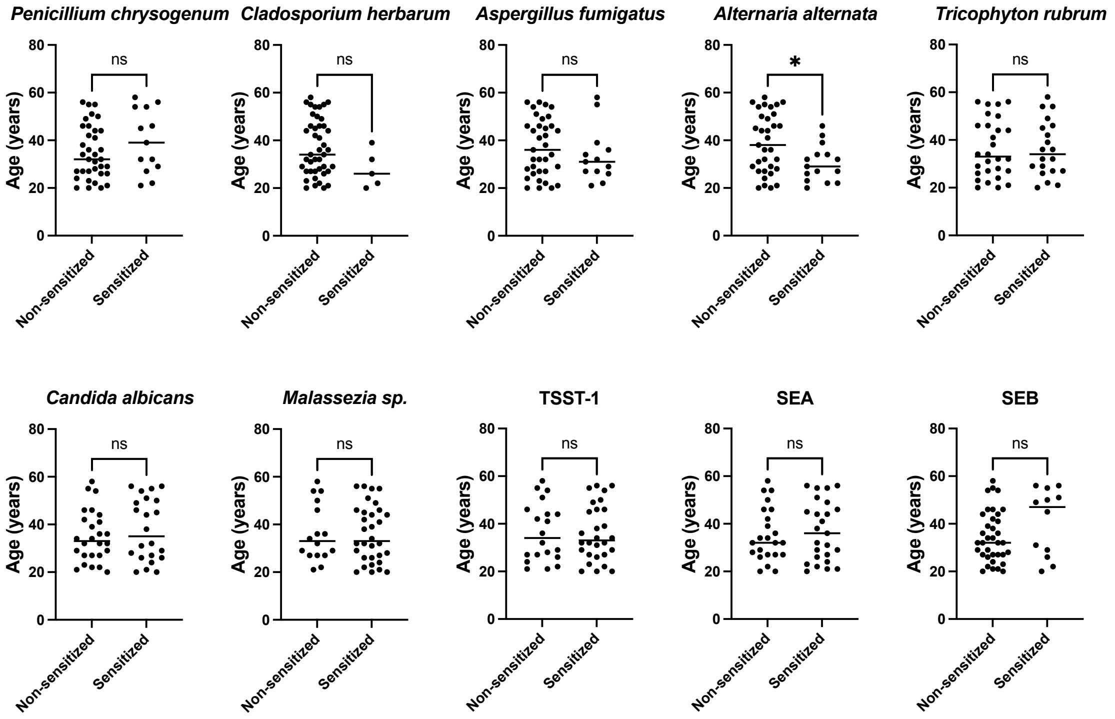

Median age (bars) was compared between the subjects sensitized and non-sensitized using Mann-Whitney U test. * $p < 0.05$.

SEA, *Staphylococcus aureus* enterotoxin A; SEB, *Staphylococcus aureus* enterotoxin B; TSST-1, toxic shock syndrome toxin-1
